# Expression of TRP Channels in Colonic Mucosa of IBS-D Patients and Its Correlation with the Severity of the Disease

**DOI:** 10.1155/2022/7294775

**Published:** 2022-05-29

**Authors:** Li Cheng, Qing-Qing Luo, Sheng-Liang Chen

**Affiliations:** ^1^Division of Gastroenterology and Hepatology, Renji Hospital, School of Medicine, Shanghai Jiao Tong University, Shanghai, China; ^2^Department of Gastroenterology, School of Medicine, Sir Run Run Shaw Hospital, Zhejiang University, Hangzhou, China

## Abstract

**Aim:**

Lots of researches have endeavored to elucidate the pathogenetic mechanism of visceral hypersensitivity in order to guide the therapy of diarrhea predominant-irritable bowel syndrome (IBS-D). Transient receptor potential (TRP) channels and their role in visceral nociception have been vastly investigated. We investigated the expression of TRP channels in IBS-D colonic biopsies and its correlation with the severity of the disease.

**Methods:**

Sigmoid biopsies were obtained from 34 IBS-D patients and 28 healthy controls (HCs). IBS-D was diagnosed according to Rome IV criteria. Their clinical parameters were assessed through questionnaires. Expression of TRPV1, TRPV4, TRPA1, TRPM2, and TRPM8 was evaluated with immunohistology staining.

**Results:**

Expression levels of TRPV1, TRPV4, and TRPA1 in the colonic mucosa of IBS-D patients were significantly higher than those in HCs (*p* < 0.05), while there was no obvious difference of TRPM2 and TRPM8 expression between IBS-D patients and HCs. In addition, the expression levels of TRPV1 and TRPA1, but TRPV4, in the colonic mucosa correlated positively with the severity of diseases (*r* = 0.6303 and 0.4506, respectively, *p* < 0.05).

**Conclusions:**

Expression of TRPV1, TRPA1, and TRPV4 in the colonic mucosa was enhanced in IBS-D patients compared with HCs with the former two correlated with the severity of the disease. TRP channels might be promising biomarkers in the diagnosis and estimate of the severity in IBS-D.

## 1. Introduction

Irritable bowel syndrome (IBS) is a disease manifested by abdominal pain and altered bowel habits, which afflicts 5 to 10% of the population in a relapsing and remitting manner [1]. According to Rome IV, the diagnosis is based on symptom analysis excluding biochemical abnormalities [2]. It is both difficult and money consuming since there is no reliable and recognized biomarker. Thus, discovery of a universally accepted biomarker is in urgent need.

Visceral hypersensitivity is one of the core pathophysiological factors of IBS [3], besides the disordered brain-gut axis [4], intestinal flora disturbance [5], or dysfunction of mucosal immunity [6]. The intestine is equipped with various ion channels and nociceptive receptors to transduce and respond to physiochemical stimuli [7]. Alteration in the expression or functioning would lead to aberrant visceral nociception. Transient receptor potential (TRP) channels are the most extensively studied ion channel family contributing to visceral hypersensitivity.

Of all TRP channels, TRPV1 is the best characterized and most studied. Enhanced expression of TRPV1 was found in the sigmoid of IBS patients [8], and the severity of the disease correlated positively with the content of TRPV1. TRPA1, as demonstrated in various literature, is an important mediator in visceral hypersensitivity [9, 10]. Evidence of its role in IBS is mainly derived from animal models. Previously, we have also ascertained its role in exacerbated mechanonociception after cold exposure in a classical animal model [11]. Unfortunately, clinical studies concerning TRPA1 still lack. It is the same case with TRPV4 [12, 13], TRPM2 [14], and TRPM8 [15, 16], as lots of preclinical studies indicated their involvement in visceral nociception.

In this study, we explored the expression of TRP channels in the colonic mucosa of IBS-D and healthy controls and investigated the relationship between the expression of TRP channels and the severity of diseases. Hopefully, it would offer more evidence of the role of TRP channels in IBS-D and help with the diagnosis and severity evaluation of the disease.

## 2. Materials and Methods

### 2.1. Human Biopsies

Thirty-four patients with IBS-D (20 males and 14 females, aged 44.53 ± 2.15 years) and 28 healthy controls (HCs) (12 males and 16 females, aged 47.43 ± 2.66 years) undergoing colonoscopy for colorectal cancer screening were enrolled in the study. IBS-D was diagnosed according to the self-completed ROME IV modular questionnaire. Clinical parameters including weight, height, and patients' abdominal pain severity measured with the visual analogue scale (VAS) [17] were collected prior to bowel preparation. All the involved subjects had no history of abdominal surgery or organic diseases. Three sigmoid mucosal samples were obtained from each subject with standard biopsy forceps and placed in 4% paraformaldehyde for 24 hours. This study has been approved by the Renji Hospital Ethics Committee. All patients and healthy controls provided written informed consent.

### 2.2. Immunohistochemistry Staining

Fixed colonic mucosal samples were dehydrated, paraffin embedded, and sectioned. Tissue sections of 4 *μ*m thickness were mounted on silicone-coated slides, deparaffinized, and heated in citrate buffer (pH 6) using high-pressure cooking for antigen retrieval. Then, the sections were incubated with primary antibodies of TRPV1 (ab3487, 1 : 50, Abcam, Cambridge, MA, USA), TRPA1 (ACC-037, 1 : 50, Alomone Labs, Jerusalem, Israel), TRPV4 (ACC-034,1 : 50), TRPM2 (ACC-043, 1 : 50), and TRPM8(ACC-049,1 : 200) at 4°C overnight. After being rinsed with phosphate buffer saline (PBS) for three times, they were incubated with goat anti-rabbit horseradish peroxidase-conjugated secondary antibody (Invitrogen, Carlsbad, CA, USA) for 30 minutes at room temperature and visualized with a diaminobenzidine-enhanced liquid substrate system (Sigma-Aldrich, St. Louis, MO, USA). For semiquantitation, captured images were analyzed with ImageJ software (National Institutes of Health).

### 2.3. Statistics

Continuous variables were expressed as mean ± SEM, and statistical analyses were performed using unpaired Student's *t*-test. Categorical data were expressed as *n* (%) and analyzed with the chi-square test or Fisher exact tests if appropriate. Correlation between the expression of TRP channels and severity of the disease was assessed by Pearson's correlation coefficient. Based on the previous study about the correlation between TRPV1 levels and pain severity [8], a sample size of at least 22 IBS-D patients was required to reach an effect size of 0.68, with a power of 95% and a 5% level of significance [18]. Data was analyzed using SPSS version 21.0 (SPSS Inc., Chicago, IL, USA). *p* < 0.05 was considered significant.

## 3. Results

### 3.1. Expression of TRPV1 Was Increased in the Colonic Mucosa of IBS-D Patients and Correlated with the Severity of the Disease

**Figure 1:**
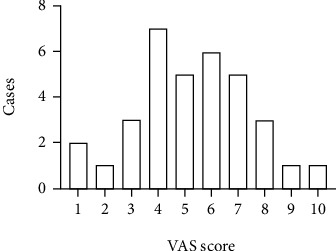
Abdominal pain severity of the IBS-D patients. VAS: visual analogue scale.

**Figure 2:**
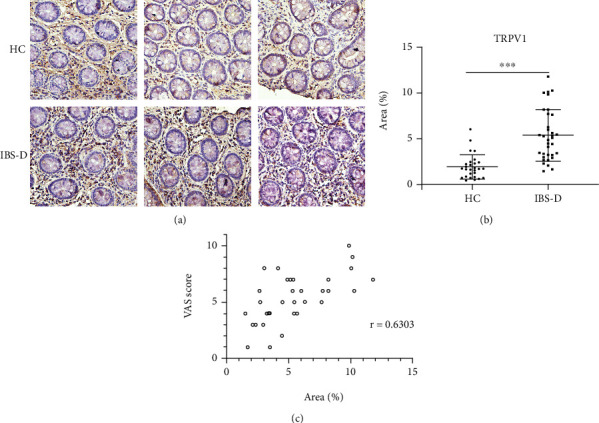
Expression of TRPV1 was increased in the colonic mucosa of IBS-D patients and correlated with the severity of the disease. (a, b) Expression of TRPV1 in the colonic mucosa of IBS-D patients was higher than that of HCs. (c) Expression of TRPV1 in the colonic mucosa correlated with the severity of IBS-D. Scale bar, 50 *μ*M. ^∗∗∗^*p* < 0.001.

To start with, no difference was detected between IBS-D and HC groups with regard to age, gender, or BMI distribution (*p* > 0.05) (Table 1). The VAS pain scores of patients were normally distributed (Figure 1). Since accumulating evidence has indicated the involvement of TRPV1 in IBS-D, we begun with TRPV1 to verify the disparate expression between HCs and IBS-D. As shown in Figures 2(a) and 2(b), the expression of TRPV1 in the IBS-D group was higher than that in the HC group (*p* < 0.0001). The correlation analysis between the expression of TRPV1 and severity of disease further revealed that IBS-D patients with higher VAS score tended to exhibit intensified expression of TRPV1 (*p* < 0.0001, *r* = 0.6303, Figure 2(c)).

### 3.2. TRPA1 and TRPV4 Expression Was Enhanced in the Colonic Mucosa of IBS-D Compared with HC

**Figure 3:**
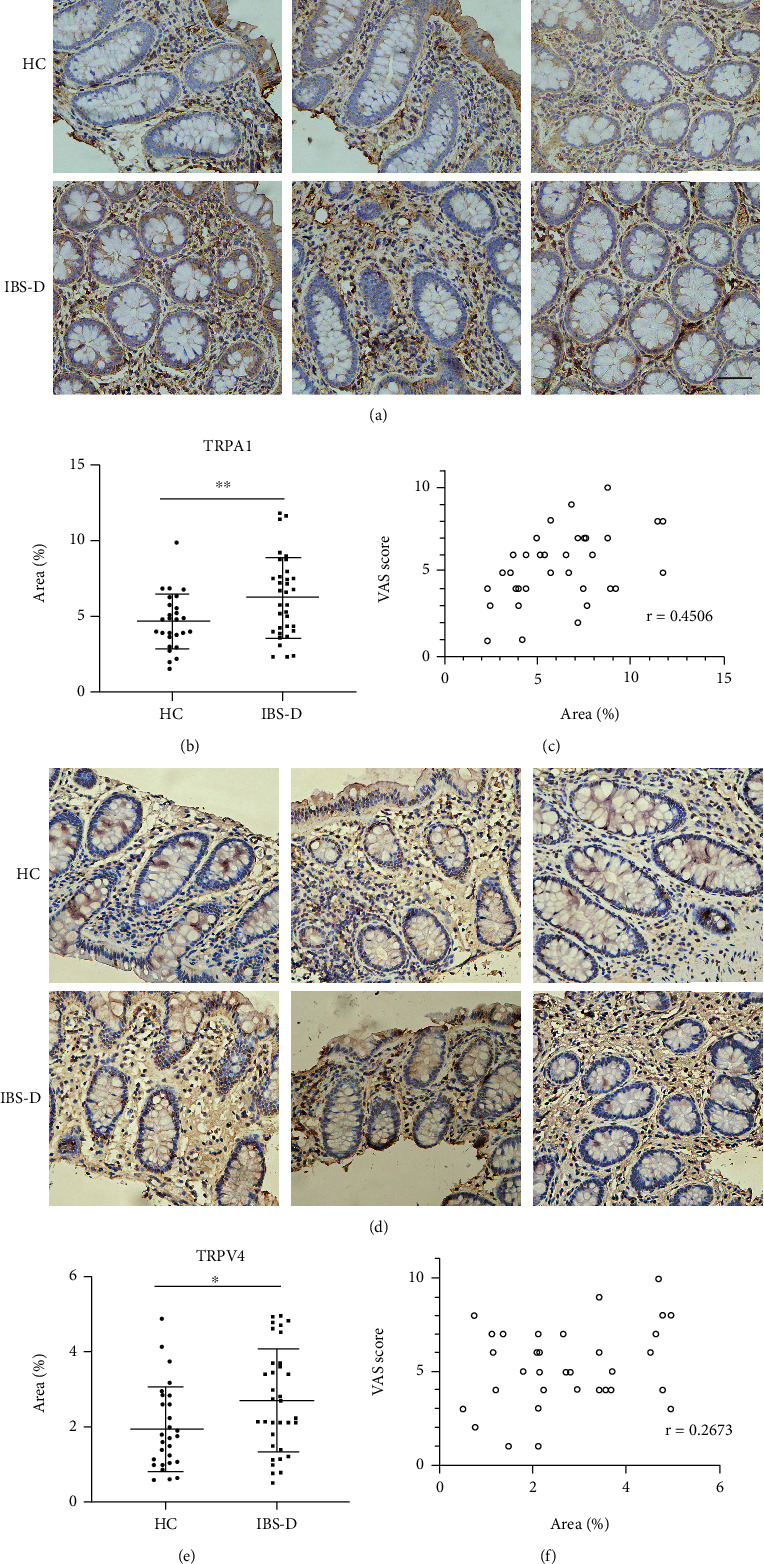
Expression of TRPA1 and TRPV4 was increased in the colonic mucosa of IBS-D patients and TRPA1 content correlated with the severity of the disease. (a, b) The expression of TRPA1 in the colonic mucosa of IBS-D patients was increased compared with HCs. (c) TRPA1 content correlated positively with the disease severity. (d, e) The expression of TRPV4 was enhanced in the colonic biopsies of IBS-D patients. (f) TRPV4 expression did not correlate with the disease severity. Scale bar, 50 *μ*M. ^∗^*p* < 0.05, ^∗∗^*p* < 0.01.

Since TRPA1 was almost exclusively expressed in TRPV1-positive neurons and they interact with each other under many circumstances [19, 20], we evaluated the expression of TRPA1 in the colonic mucosa. It was shown that expression of TRPA1 was enhanced in the biopsies of the IBS-D group compared with HCs (*p* = 0.008, Figures 3(a) and 3(b)). Further analysis demonstrated a slightly weaker but significant correlation between the expression of TRPA1 and the severity of disease (*p* = 0.0075, *r* = 0.4506, Figure 3(c)). TRPV4 was another TRP channel with enhanced expression in the colonic mucosa of IBS-D patients (Figures 3(d) and 3(e)). However, no correlation between the expression level and severity of the disease was found (*p* > 0.05).

### 3.3. TRPM2 and TRPM8 Expression Did Not Differ between IBS-D and HC

**Figure 4:**
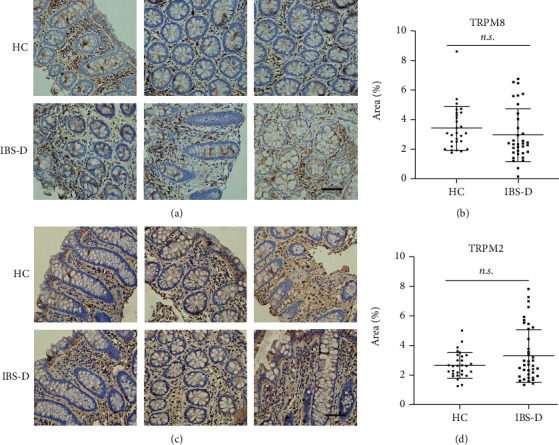
Expression of TRPM2 and TRPM8 did not differ between IBS-D patients and HCs. (a, b) No difference in the expression of TRPM8 was found between IBS-D and HC groups. (c, d) No difference in the expression of TRPM2 was found between IBS-D and HC groups. Scale bar, 50 *μ*M.

Our previous study has found the role of TRPM8 in visceral hypersensitivity [15]. The immunostaining of TRPM8, however, showed no difference between IBS-D and HC (*p* > 0.05, Figures 4(a) and 4(b)). It was the same case with TRPM2 (*p* > 0.05, Figures 4(c) and 4(d)).

## 4. Discussion

Relief of visceral pain is an unmet need in clinical practice of IBS-D treatment. Mounting evidence indicated the involvement of TRP channels in visceral hypersensitivity, one of the core pathophysiological mechanisms. Except for TRPV1, most studies about TRP channels focused on preclinical animal models and the results varied. Our present study is aimed at comparing the expression of TRP channels in the colonic mucosa of IBS-D patients and HCs and correlation between the expression and the severity of the disease.

The role of TRPV1 in IBS has been extensively investigated [21, 22]. In our study, we verified a similar phenomenon that expression of TRPV1 was enhanced in the colonic mucosa of IBS-D patients compared with HCs and correlated with the severity of the disease [8, 21], further supporting TRPV1 as a valuable target in coping with visceral pain.

Previous researches about the role of TRPA1 in visceral hypersensitivity were mainly based on preclinical studies [23, 24]. In Kun's study, the mRNA level of TRPA1 was increased in the biopsies of active IBD patients rather than in the quiescent state [25], indicating its involvement in acute pain perception. In our study, TRPA1 expression was increased in the colonic mucosa of IBS-D patients and the expression level positively correlated with the severity of the disease. According to some researchers' opinion, TRPA1 was sensitized rather than overexpressed in IBS [26]. The different results may be due to the subtype of IBS patients included in the study. Even though the results concerning TRPA1 were promising, more clinical studies are warranted since the sample size in this study was comparatively small.

Activation of TRPV4 triggers visceral hypersensitivity based on mounting evidence. In one study, it was found that 5,6-EET (an endogenous TRPV4 agonist), but not TRPV1 or the TRPA1 agonist, was increased in the biopsies of IBS patients compared with HCs and the concentration correlated with abdominal pain [27]. According to McGuire et al., human serosal nociceptor mechanosensitivity was attenuated by TRPV4 antagonist, HC067047 [28], which is evidenced by the role of TRPV4 in visceral hypersensitivity. In our study, the expression of TRPV4 in the colonic mucosa was increased in IBS-D groups. However, no relationship was found between the expression level and disease severity.

TRPM2 and TRPM8 were the relatively less-studied TRP channels. We have previously found that either altered distribution or expression of TRPM8 in sensory neurons under different circumstances contributed to visceral hypersensitivity [15]. For TRPM2, its expression was enhanced in the distal colon of a TNBS-colitis rat model and administration of TRPM2 antagonist or TRPM2 deficiency-attenuated visceromotor response to colorectal distension [14]. Unfortunately, in this study, we did not find altered expression of these two TRP channels in IBS-D patients compared with HCs. There were studies suggesting upregulated TRPM8 as a protective factor against inflammatory mediators [16, 29, 30]. With divergent opinion towards the role of TRPM8 and TRPM2, more preclinical and clinical studies are needed to reach a consensus.

The strength of this study is that we compare the expression of TRP channels in IBS-D, a specific subtype of IBS, and HCs, since different subtypes of IBS may have different pathophysiological mechanisms. Besides, we explored the relationship between the expression of TRP channels and the severity of the disease, which may offer insights into the diagnosis and severity measurement of IBS-D. However, the shortcomings of this study are obvious. First, the sample size was rather small. Secondly, a lot of clinical parameters are missing, e.g., food pattern, psychological factors, and stool frequency.

## 5. Conclusions

In conclusion, TRPV1 is a promising factor in the diagnosis and severity evaluation of IBS-D. It may become an intervenable target in dealing with IBS-D in the future. More preclinical and clinical studies may help verdict the role and relationship between TRP channels and IBS-D.

## Figures and Tables

**Table 1:** Patient demographics.

		HC	IBS-D	*p* value
Case, *n*		28	34	
Age, mean ± SEM		47.43 ± 2.66	44.53 ± 2.15	0.820
Gender, *n* (%)	Female	16 (57.1%)	14 (41.2%)	0.211
	Male	12 (42.9%)	20 (58.8%)	
BMI, *n* (%)	Thin	4 (14.3%)	3 (8.8%)	0.921
	Normal	16 (57.1%)	21 (61.8%)	
	Overweight	7 (25.0%)	9 (26.5%)	
	Obese	1 (3.6%)	1 (2.9%)	

BMI: body mass index (kg/m^2^, calculated as weight/height^2^), thin < 18.5, 18.5 ≤ normal < 24, 24 ≤ overweight < 28, and obese ≥ 28.

## Data Availability

The data used to support the findings of this study are available from the corresponding author upon request.
